# Supplementary material for: Infrared nanoscopy and tomography of intracellular structures
Source: Commun Biol. 2021 Nov 30;4:1341. doi: 10.1038/s42003-021-02876-7 (PMC8633277; doi:10.1038/s42003-021-02876-7)
Supplement: Supplementary file 2 — Supplementary Information [file 42003_2021_2876_MOESM2_ESM.pdf]

Supplementary Information

Infrared Nanoscopy and Tomography of Intracellular Structures

Katerina Kanevche[1], David J. Burr[2], Dennis J. Nürnberg[3], Pascal K. Hass[4], Andreas Elsaesser[2], Joachim Heberle[1]

[1]Freie Universität Berlin, Department of Physics, Experimental Molecular Biophysics, Arnimallee 14, 14195, Berlin, Germany

[2]Freie Universität Berlin, Department of Physics, Experimental Biophysics and Space Sciences, Arnimallee 14, 14195, Berlin, Germany

[3]Freie Universität Berlin, Department of Physics, Biochemistry and Biophysics of Photosynthetic Organisms, Arnimallee 14, 14195, Berlin, Germany

[4]Freie Universität Berlin, Department of Veterinary Medicine, Institute of Veterinary Anatomy Koserstr. 20, 14195, Berlin, Germany

Corresponding authors:

Joachim Heberle joachim.heberle@fu-berlin.de

Andreas Elsaesser a.elsaesser@fu-berlin.de

Supplementary Information

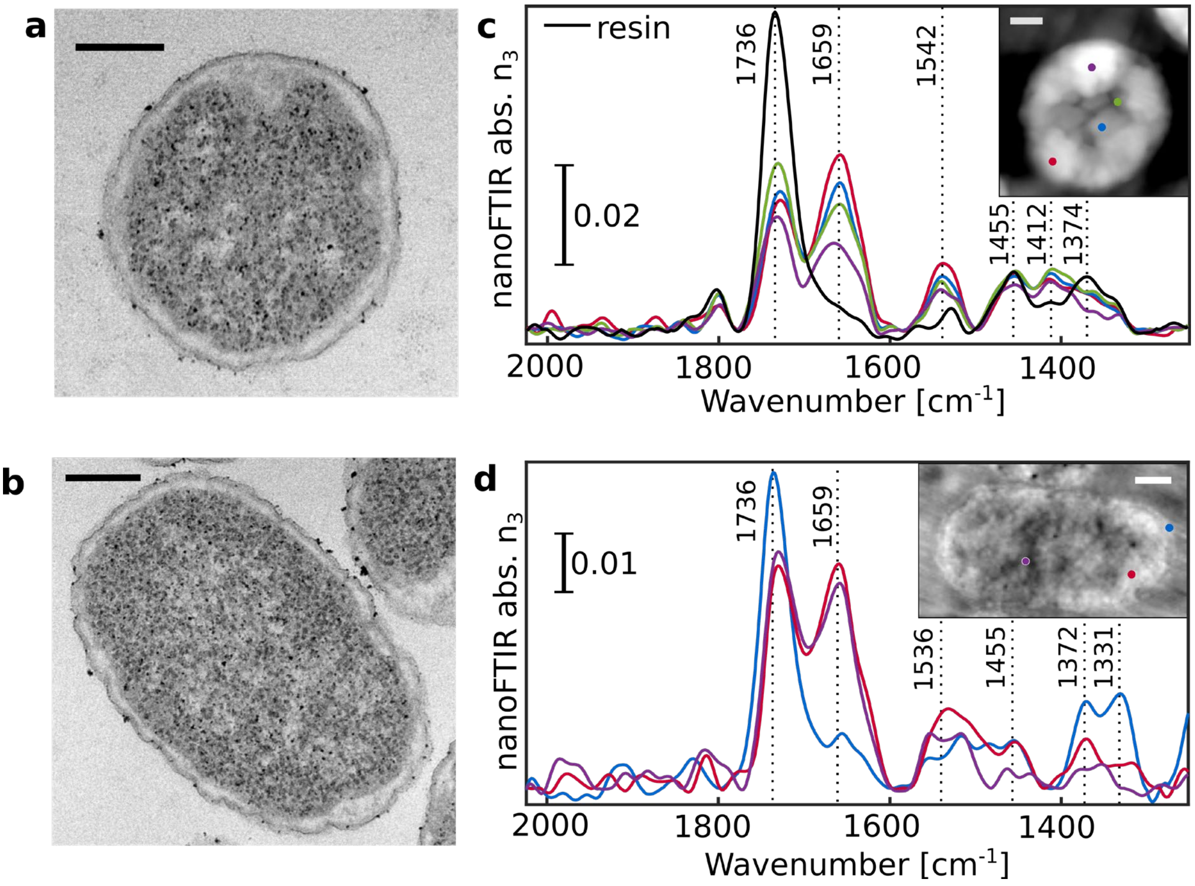

Supplementary Figure 1 *E. coli* cross sections imaged by TEM (**a** and **b**) and AFM (insets **c** and **d**). Scale bars equal 200 nm. NanoFTIR absorption spectra (**c** and **d**) were acquired at the locations marked on the AFM images.

TEM imaging (Figure S1 a and b) of *E. coli* is highly comparable to AFM topography (Figure S1 c and d, inserts). Similar cell diameters (~700 nm), lengths (1.5 µm), shapes and homogenous morphologies can be observed. Resin bands (~1740 cm^{-1}) are present in all spectra, indicating strong resin penetration throughout the cells. In addition to the resin peak and characteristic amide I (1658 cm^{-1}) and amide II (1542 cm^{-1}) absorption bands, spectroscopic analysis of *E. coli* revealed several absorption bands in the lower wavenumber region (Figure S1c and d); absorption at ~1450 cm^{-1} is typical for C-H deformation of CH and CH_2, and ~1350 cm^{-1} corresponds to C-H deformation in CH_3 groups. The spectrum measured on the cell edge (Figure S1 d, blue) shows absorption bands characteristic of both the resin (~1740 cm^{-1}) and peptides (1659 cm^{-1}), for instance peptidoglycan [1]. This spectrum shows additional absorption

around 1370 cm^{-1} that can be assigned to the amide III bands, due to the complex sugars content in peptidoglycans.

With the exception of intensity differences between amide I and II bands, there are only minute variations in local protein content. This homogeneity in intracellular chemical composition is not surprising, considering that, *E. coli* is a prokaryotic organism and does not have any membrane-bound organelles.

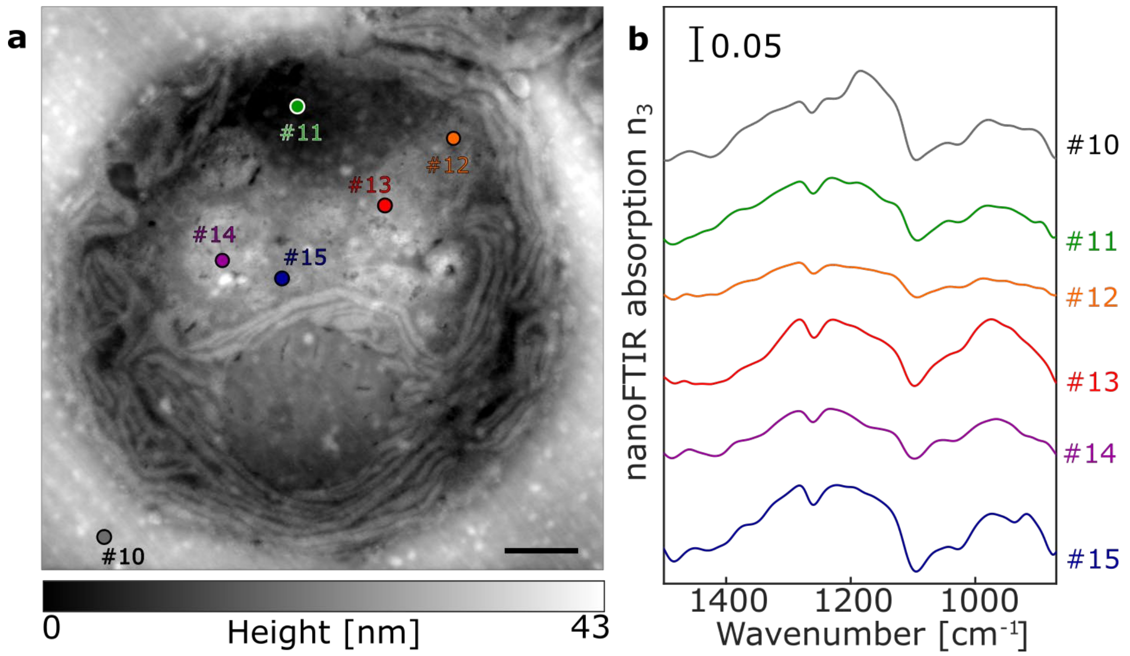

Supplementary Figure 2 *C. reinhardtii* cross section imaged by AFM topography (**a**) with the scale bar showing 1 µm. NanoFTIR absorption spectra (**b**) were acquired at the locations marked on the AFM image.

To complement the nanoFTIR measurements performed on *C. reinhardtii* (Figure 2), several additional spectra focusing on the low-wavenumber region (down to ~1000 cm^{-1}) were measured. These were recorded on the surrounding resin (#10), the nucleus (#11) and in the cytoplasmic region (#12, #13, #14 and #15). C-O stretching vibration typically absorb in the spectral region around 1280 cm^{-1}, therefore the spectral features in the spectra in the cytoplasmic area point to resin absorption. In the spectrum measured on the nucleus, a peak at around 1240 cm^{-1} can be assigned to the P=O asymmetric stretch of the phosphoryl group or DNA-base sugar vibrations [2]. Near-field absorption in this spectral range is evident also in the rest of the spectra, which could originate from C-C-O stretching vibrations in the

resin. The source of the absorption features in the range 1190-1120 cm^{-1} and at around 980 cm^{-1} is assigned to absorption of complex sugar modes [3]. Additionally, C-O stretching vibrations can also be assigned around 1155 cm^{-1} and therefore is observed both on the cellular surface and on the resin area [4].

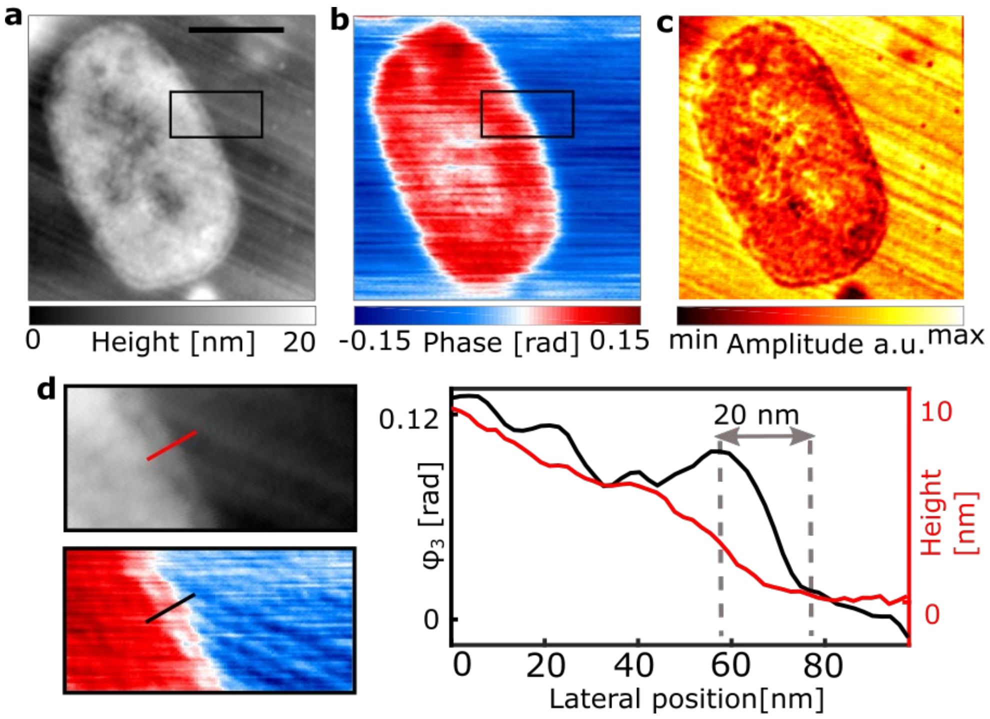

Supplementary Figure 3 Imaging of a single *E. coli* cell, visualized by AFM topography (**a**) with the scale bar showing 1 µm, sSNOM phase imaging (**b**) and sSNOM amplitude (**c**) at 1658 cm^{-1}. (**d**) AFM and phase image of the marked area in (a) and (b) with height profile (red curve) and sSNOM phase profile (black curve).

AFM topography, and sSNOM phase and amplitude imaging at 1658 cm^{-1} each reveal a non-homogenous distribution of intracellular material (Figure S3a – c). In addition to the cellular envelope showing less amide I absorption than within the cell (Figure S3b), the cytoplasmic region contains several regions that correspond to a reduction in AFM height, and reduced absorption and increased scattering at 1658 cm^{-1}. Furthermore, sSNOM phase absorption reveals a small region of increased amide I density in the top right of the cell. This heterogenous distribution indicates that this technique can detect locally isolated protein regions within a single cell.

The line profile reveals 20 nm lateral resolution, whilst also demonstrating the difference in detection limitation of AFM and sSNOM phase imaging (Figure S3d).

	1738 cm^{-1}	1655 cm^{-1}	1540 cm^{-1}	1238 cm^{-1}	1155 cm^{-1}
Pyrenoid		Amide I	Amide II		
Nucleus		Pyrine/pyridine ring mode; Amide I	Pyrine/pyridine ring mode; Amide II		
Nucleolus		Pyrine/pyridine ring mode; DNA base-sugar vibrations; Amide I	Pyrine/pyridine ring mode; DNA base-sugar vibrations; Amide II	Asymmetric O-P-O stretching vibration	
Cajal body		Pyrine/pyridine ring mode; DNA base-sugar vibrations; Amide I	Pyrine/pyridine ring mode; DNA base-sugar vibrations; Amide II		
Thylakoid membrane	C=O (chlorophyll)	Amide I	Amide II		C-O-C stretch ester
Cell wall	C=O (lipids)	Amide I	Amide II		C-O-C stretch ester; complex sugar modes

Supplementary Table 1 Summary of subcellular components of *C. reinhardtii* that show contrast at different wavenumbers. The assignment to cellular structures at 1738 cm^{-1} (in parentheses) is ambiguous due to resin absorption.

Supplementary References

1 Huang, K. C., Mukhopadhyay, R., Wen, B. N., Gitai, Z. & Wingreen, N. S. Cell shape and cell-wall organization in Gram-negative bacteria. *P Natl Acad Sci USA* **105**, 19282-19287, doi:10.1073/pnas.0805309105 (2008).

2 Han, Y., Han, L., Yao, Y., Li, Y. & Liu, X. Key factors in FTIR spectroscopic analysis of DNA: the sampling technique, pretreatment temperature and sample concentration. *Analytical Methods* **10**, 2436-2443, doi:10.1039/c8ay00386f (2018).

3 Wiercigroch, E. *et al.* Raman and infrared spectroscopy of carbohydrates: A review. *Spectrochimica Acta Part A: Molecular and Biomolecular Spectroscopy* **185**, 317-335, doi:https://doi.org/10.1016/j.saa.2017.05.045 (2017).

4 González, M. G., Cabanelas, J. C. & Baselga, J. in *Infrared Spectroscopy - Materials Science, Engineering and Technology* Ch. Chapter 6, (IntechOpen, 2012).
