# Supplementary material for: Infrared nanoscopy and tomography of intracellular structures
Source: Commun Biol. 2021 Nov 30;4:1341. doi: 10.1038/s42003-021-02876-7 (PMC8633277; doi:10.1038/s42003-021-02876-7)
Supplement: Supplementary file 3 — Description of Additional Supplementary Files [file 42003_2021_2876_MOESM3_ESM.pdf]

Description of Additional Supplementary Files

File name: Supplementary Movie 1.
Description: 3D reconstruction of ten sSNOM phase images of *C. Reinhardtii*, recorded at 1656 cm^{-1}.
